# The Influence of Oxidation on the Magnetic, Electrical, and Mechanical Properties of Co_40_Fe_40_Yb_20_ Films

**DOI:** 10.3390/ma15238675

**Published:** 2022-12-05

**Authors:** Wen-Jen Liu, Yung-Huang Chang, Chia-Chin Chiang, Yuan-Tsung Chen, Ying-Hsuan Chen, Hui-Jun You, Te-Ho Wu, Shih-Hung Lin, Po-Wei Chi

**Affiliations:** 1 Department of Materials Science and Engineering, I-Shou University, Kaohsiung 84001, Taiwan; 2 Bachelor Program in Interdisciplinary Studies, National Yunlin University of Science and Technology, 123 University Road, Section 3, Douliou, Yunlin 64002, Taiwan; 3 Department of Mechanical Engineering, National Kaohsiung University of Science and Technology, 415 Chien Kung Road, Kaohsiung 80778, Taiwan; 4 Graduate School of Materials Science, National Yunlin University of Science and Technology, 123 University Road, Section 3, Douliou, Yunlin 64002, Taiwan; 5 Department of Electronic Engineering, National Yunlin University of Science and Technology, 123 University Road, Section 3, Douliou, Yunlin 64002, Taiwan; 6 Institute of Physics, Academia Sinica, Nankang, Taipei 11529, Taiwan

**Keywords:** annealed Co_40_Fe_40_Yb_20_ thin films, oxidation effect, X-ray diffraction (XRD), low-frequency alternating current magnetic susceptibility (χ_ac_), adhesion, electrical characteristics, mechanical property

## Abstract

A typical body-centered cubic (BCC) CoFe(110) peak was discovered at approximately 2θ = 44.7°. At 2θ = 46°, 46.3°, 47.7°, 55.4°, 54.6°, and 56.4°, the Yb_2_O_3_ and Co_2_O_3_ oxide peaks were visible in all samples. However, with a heat treatment temperature of 300 °C, there was no typical peak of CoFe(110). Electrical characteristics demonstrated that resistivity and sheet resistance reduced dramatically as film thickness and annealing temperatures increased. At various heat treatments, the maximum hardness was 10 nm. The average hardness decreased as the thickness increased, and the hardness trend decreased slightly as the annealing temperature was higher. The highest low-frequency alternative-current magnetic susceptibility (χ_ac_) value was discovered after being annealed at 200 °C with 50 nm, and the optimal resonance frequency (f_res_) was discovered to be within the low-frequency range, indicating that the Co_40_Fe_40_Yb_20_ film can be used in low-frequency applications. The maximum saturation magnetization (Ms) was annealed at 200 °C for 50 nm. Thermal disturbance caused the Ms to decrease as the temperature reached to 300 °C. The results show that when the oxidation influence of as-deposited and thinner films is stronger than annealing treatments and thicker thickness, the magnetic and electrical properties can be enhanced by the weakening peak of the oxide, which can also reduce interference.

## 1. Introduction

CoFe alloys are frequently used in a wide range of magnetic devices, including sensors, actuators, magnetic read heads, and magnetic random-access memory (MRAM) [1,2,3]. Magnetic tunnel junction (MTJ) is the primary memory component of MRAM (MTJ). It is made of a barrier layer that acts as insulation and two layers of ferromagnetic metal [4,5,6,7,8]. The characteristics of the MTJ are significantly influenced by the kind, structure, and method used to manufacture the ferromagnetic layer [9,10]. The ideal soft magnetic material for the ferromagnetic layer has high saturation magnetization (Ms), high Curie temperature (Tc), low coercivity (Hc), high permeability (μ), and low magnetostriction (λs) in order to produce magnetization inversion at the lowest possible energy cost [11]. CoFe is an excellent candidate because of its soft magnetic properties, as well as its high Ms and Tc, which make it suitable for high-temperature applications [12]. By adjusting the Co to Fe ratio, it is possible to modify the magnetic properties for a specific application [13]. However, the CoFe alloy does not have a low Hc, and increasing the annealing temperature causes the degeneration of magnetic anisotropy faults, making it difficult to meet the magnetic equipment used at high temperatures. A third element is added to improve the thermal stability of the CoFe alloy.

High Ms, Tc, and magnetic anisotropy field (Ha) are common properties of rare earth magnetic materials. Yb is a rare earth element with distinct optical and magnetic properties as well as an incomplete 4f electronic state [14]. A superior laser material is Yb^3+^-doped YAG fluorescent powder [15]. CoFeYb is a novel and vital soft magnetic material with numerous applications in MRAM and sensors. It is also compatible with other layers in double-layer and multi-layer systems and can be used as a free or pinned layer to combine with magnetic processes. Its performance is especially vulnerable to high temperatures and operating temperatures (RT). As a result, studying the performance of CoFeYb films in their as-deposited and annealed states is worthwhile. Few studies have been conducted on the magnetic, surface energy, and mechanical properties of as-deposited and annealed CoFeYb films. The oxidation was roughly the same at all thicknesses, according to the X-ray diffraction (XRD) measurements, and the proportion of oxides increased in thinner films. Furthermore, it is observed that oxide formation has a considerable impact on hardness, magnetic, electrical, and surface energy.

## 2. Materials and Methods

CoFeYb was sputtered onto a Si(100) substrate using a magnetron sputtering direct current (DC) method with a power of 50 W under the following four conditions: (a) as-deposited films were kept at room temperature (RT), (b) annealed at 100 °C for 1 h, (c) annealed at 200 °C for 1 h, and (d) annealed at 300 °C for 1 h. The chamber base pressure was 5 × 10^−7^ Torr and the operating pressure for Ar was 2 × 10^−3^ Torr. The target composition of the CoFeYb alloy is 40% Co, 40% Fe, and 20% Yb. The ex-situ annealed condition pressure for a specific Ar gas was 2.5 × 10^–3^ Torr. The crystal structure was examined using grazing incidence X-ray diffraction (GIXRD) patterns obtained with CuKα1 (PAN analytical X’pert PRO MRD) and a low angle diffraction incidence of roughly two degrees. The surface energy is determined by measuring the contact angle with a contact angle measuring tool (CAM-110) [16,17,18]. The electrical properties are detected by four-point probe measurement. In-plane low-frequency alternate-current magnetic susceptibility (χ_ac_) and hysteresis loop were studied using alternating gradient magnetometer (AGM) and χ_ac_ analyzer (XacQuan). The continuous stiffness measurement (CSM) technique and the MTS Nano Indenter XP with a Berkovich tip were used to test the hardness. Once the load has been reduced to 10% of the maximum load, remove the indent from the surface at the same rate. Measurement should be repeated ten times for each sample with the probe. The indentation load is multiplied by 40 stages, with each step’s indentation depth being recorded. In order to produce more precise data, six indentations from each sample were evaluated, and the standard deviations were averaged. Because the film is so thin, even with a little load, the nanoindentation measurement experiment cannot escape the substrate effect. Nanoindentation testing revealed hardness (H) and lower elastic modulus (Er). To compute the mechanical properties, Oliver and Pharr’s approach was used. This method involves calculating the slope of the unloading curve in order to determine the stiffness (S) of the film. The relationship between Er and S can be explained in one way by the following: [19,20]:

Er = π^1/2^/2 = S/A^1/2^, (1)

where A is the area of contact under maximum load. The same area of contact value and the maximum load (Pmax) are used to determine the hardness values:

H = Pmax/A.(2)

## 3. Results

### 3.1. Analysis of X-ray Diffraction, Grain Size, and Full Width of Half Maximum

**Figure 1:**
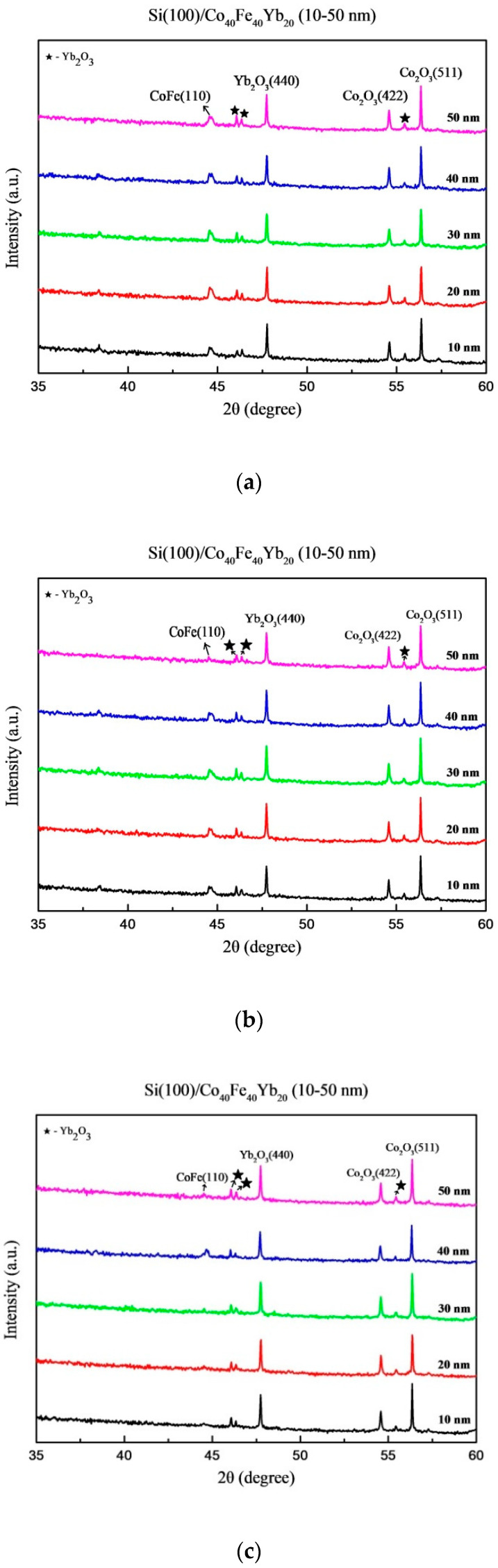

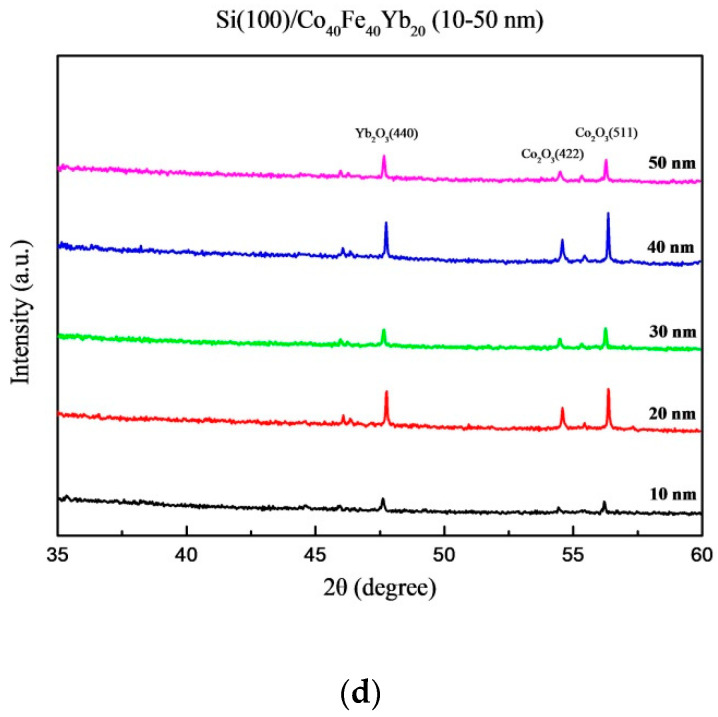
Thin films made of Co_40_Fe_40_Yb_20_ with X-ray diffraction patterns. (**a**) As-deposited, (**b**) post-annealing at 100 °C, (**c**) post-annealing at 200 °C, and (**d**) post-annealing at 300 °C.

Figure 1 displays the XRD patterns of thin films with as-deposited and annealed thicknesses ranging from 10 nm to 50 nm. Figure 1a depicts the patterns of thin films formed at RT, while Figure 1b–d depicts the patterns of post-annealing treatments. The body-centered cubic (BCC) CoFe(110) peaks were observed at around 2θ = 44.7°, indicating that the Co_40_Fe_40_Yb_20_ thin films were crystallized, except when annealed at 300 °C. The Yb_2_O_3_ and Co_2_O_3_ oxide peaks appeared at 2θ = 46°, 46.3°, 47.7°, 55.4°, 54.6°, and 56.4° in all samples. Oxidation peaks were formed as a result of the sputtering system, natural oxides on the Si (100) substrate, and oxygen contamination on the sputtering target [21]. With increasing thickness and annealing temperature, all oxide peak intensities decreased. All thicknesses had the same level of oxidation, and as thicknesses became thinner, the proportion of oxides increased. As a result, as the thickness increased, the intensity of the oxide peaks gradually decreased. The weakening oxide peak may have beneficial effects on the magnetic and electrical properties by reducing interference. The variation in the average lattice parameter determines the strain in CoFe films. This lattice strain might be either tensile or compressive. The lattice strain was calculated using the relation [22]:

Strain (%) = Δa/*a* × 100%, (3)

where *a* is the lattice parameter (for CoFe lattice parameter, *a* = 0.285 nm) [23]. The CoFe(110) film has a BCC structure, whereas Si(100) has a face-centered cubic (FCC) structure, which causes substantial lattice strain or interfacial stress and produces CoFe(110) peak deterioration at thicker thicknesses and higher annealed temperatures [24].

XRD was used to determine the full width at half maximum (FWHM), which was then used to calculate grain size using Scherrer’s equation.

Scherrer’s formula is [25]:

D = Kλ/βcosθ.(4)

**Figure 2:**
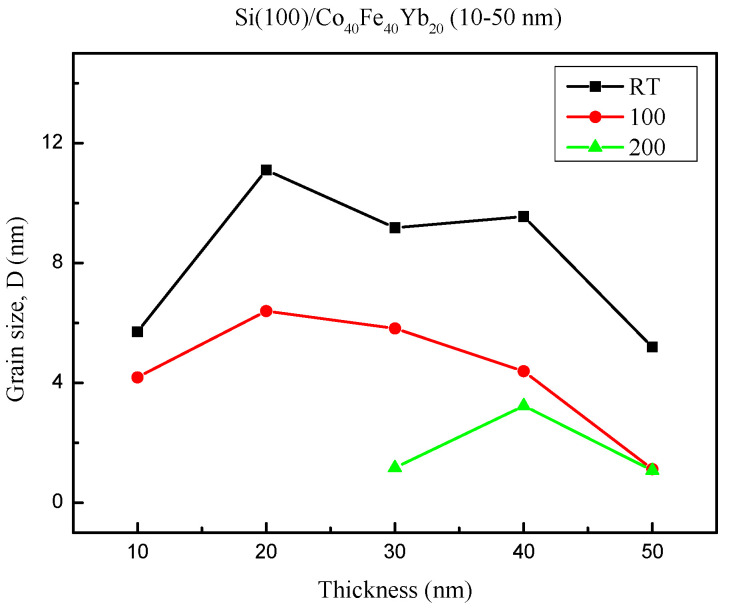
Grain size of Co_40_Fe_40_Yb_20_ thin films with CoFe (110) diffracted peak.

In the formula, k (0.89) denotes Scherrer’s constant; λ is the X-ray wavelength of the Cu Kα1 line; B denotes the FWHM diffraction CoFe(110) peak; and θ is the half-angle of the diffraction peak. Figure 2 shows the average grain sizes calculated from half of the maximum FWHM of the CoFe(110) peak under four different conditions. The CoFe(110) feature peak does not appear at an annealed temperature of 300 °C and the grain size cannot be calculated. The experimental results show that the annealed temperature increases and the grain size is reduced due to insufficient time at low-temperature annealing treatment [26].

**Figure 3:**
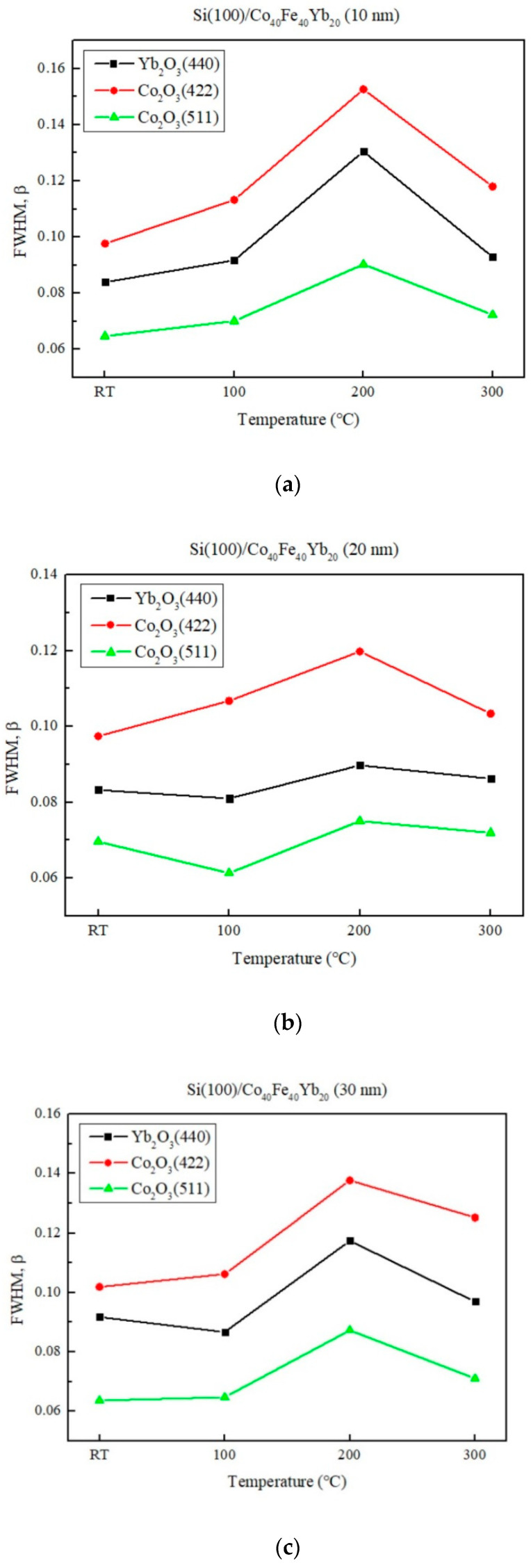

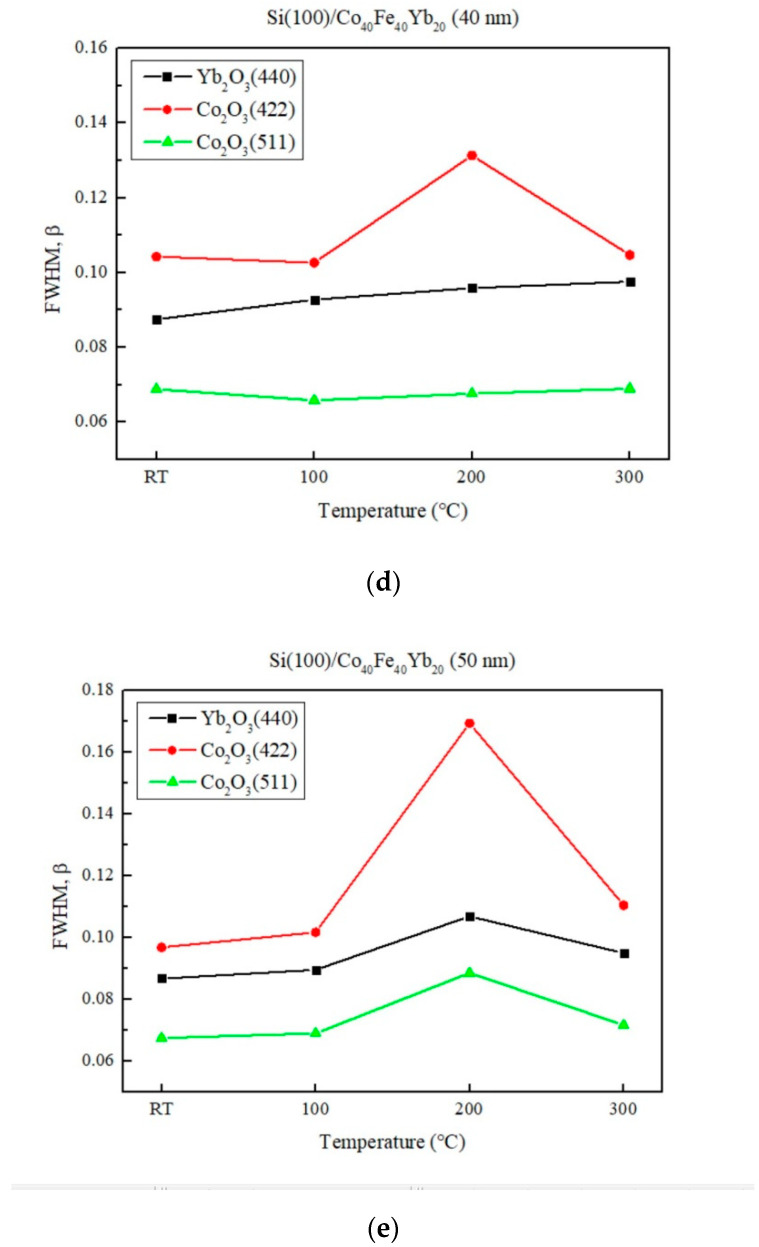
FWHM of the oxide peaks in Co_40_Fe_40_Yb_20_ films under different conditions. (**a**) 10 nm, (**b**) 20 nm, (**c**) 30 nm, (**d**) 40 nm, and (**e**) 50 nm.

To demonstrate that the more oxidation effect of thinner thickness and lower annealed temperature is stronger than thicker thickness and higher annealed temperature, the FWHM of oxide diffracted peaks is shown in Figure 3a–e. From this result, it indicates that the FWHM of thinner thickness and lower annealed temperature is weaker than thicker thickness and higher annealed temperature, suggesting that the oxidation effect is more apparent in thinner thickness and lower annealed temperatures. The magnetic, electrical, surface energy, and hardness of Co_40_Fe_40_Yb_20_ films are significantly influenced by their degree of oxidation.

### 3.2. Surface Energy and Adhesion Analysis

**Figure 4:**
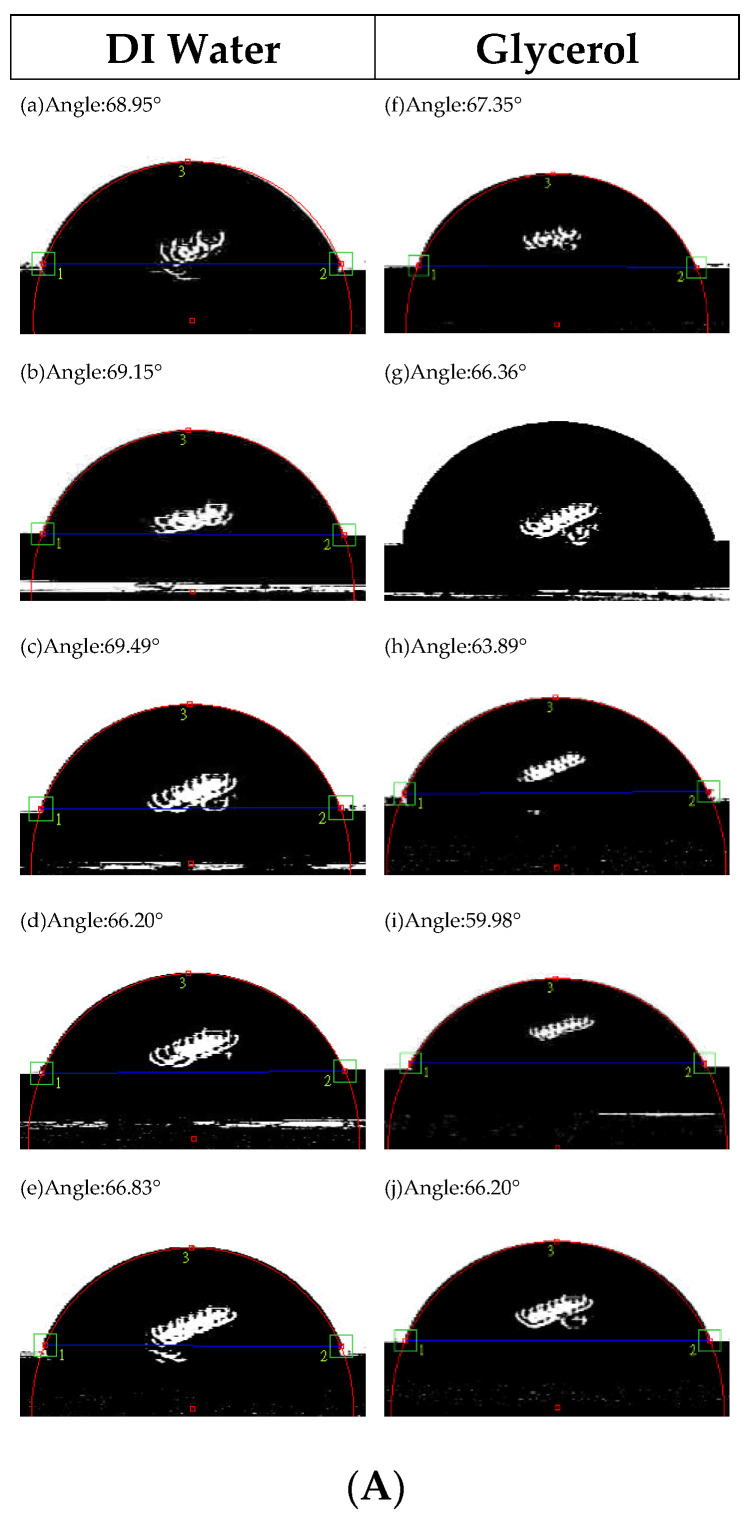

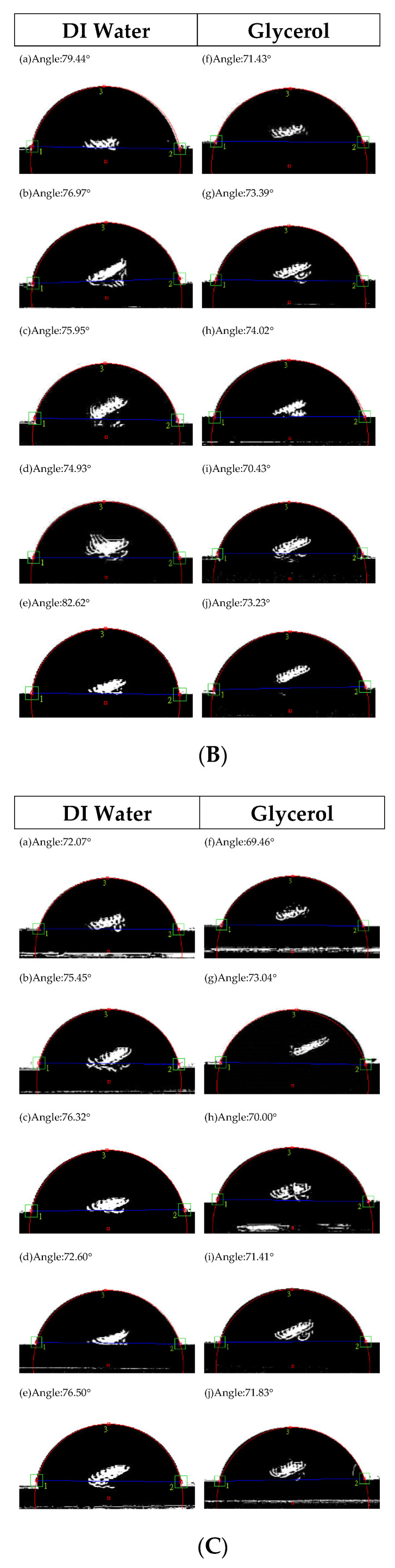

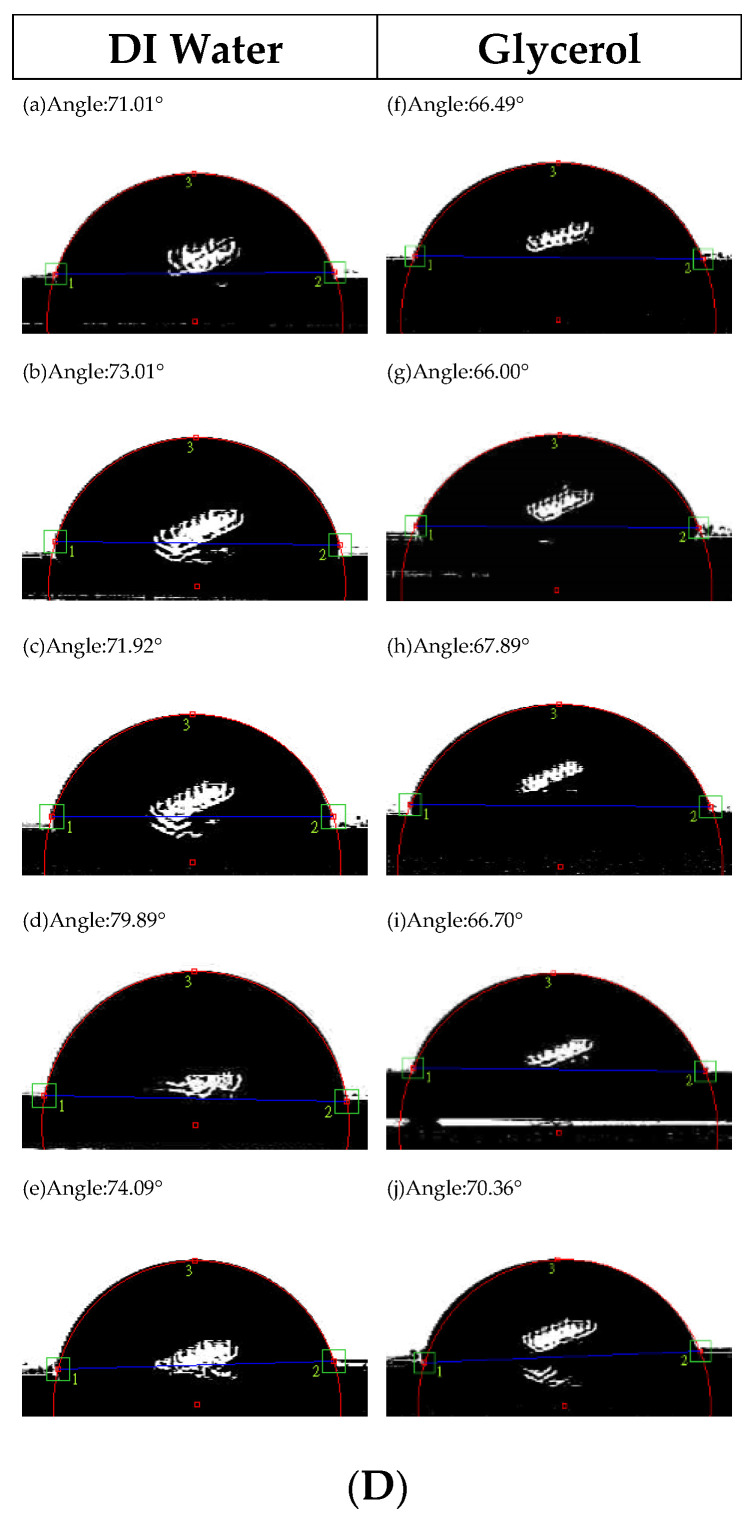
Contact angles of Co_40_Fe_40_Yb_20_ thin films under four conditions: (**A**) RT, (**B**) after annealing at 100 °C, (**C**) after annealing at 200 °C, and (**D**) after annealing at 300 °C with DI water: (**a**) 10 nm, (**b**) 20 nm, (**c**) 30 nm, (**d**) 40 nm, and (**e**) 50 nm. Glycerol: (**f**) 10 nm, (**g**) 20 nm, (**h**) 30 nm, (**i**) 40 nm, and (**j**) 50 nm.

Figure 4A–D show the contact angles (θ) of Co_40_Fe_40_Yb_20_ films under four conditions. The contact angles of the films were examined using DI water and glycerol. In particular, the Co_40_Fe_40_Yb_20_ films were observed to have contact angles that were always less than 90° and the drops were nearly spherical, demonstrating the films had good hydrophilicity and wettability. Surface energy and adhesion are significant factors because the Co_40_Fe_40_Yb_20_ film can be used as a seed or buffer layer. The contact angle decreases due to significant liquid absorption when the surface energy is high. Using Young’s equation and the contact angle, the surface energy is computed [16,17,18].

**Figure 5:**
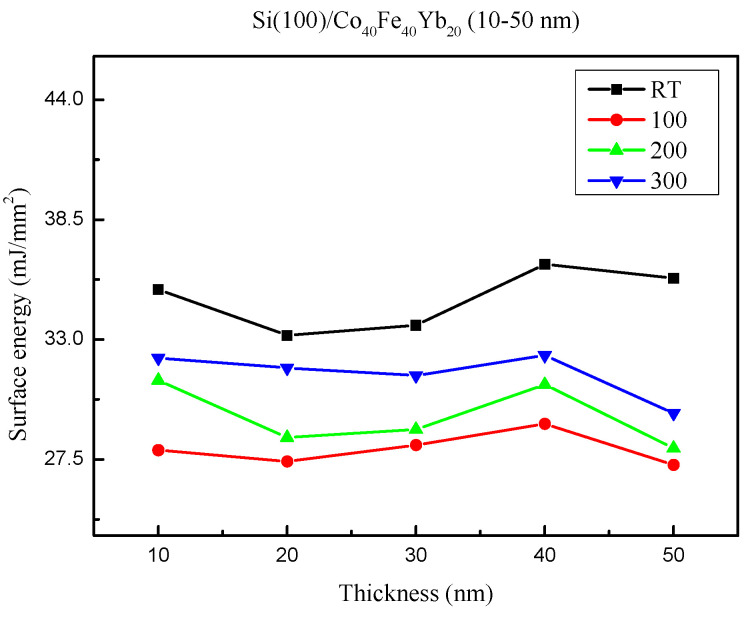
Surface energy of Co_40_Fe_40_Yb_20_ thin films.

Figure 5 shows the surface energy under all conditions. The surface energy ranged from 27.25 mJ/mm^2^ to 36.45 mJ/mm^2^. The strongest adhesion occurred when the films had a larger surface energy. This study mostly indicates that more oxide layers form on thinner film surfaces, resulting in smaller contact angles and higher surface energies, and that these conditions favor stronger CoFe(110) crystallization. Additionally, the maximal surface energy is reached at a thickness of 40 nm. These findings imply that the relationship between surface energy and thickness was concave-up, with 40 nm being the crucial thickness. Furthermore, strong adhesion is correlated with high surface energy. Thus, it can be reasonably concluded in this study that the as-deposited condition has stronger crystallization. The weakest crystallization implies that more impurities or defects exist by annealing treatment, reducing surface energy.

### 3.3. Electrical Examination

**Figure 6:**
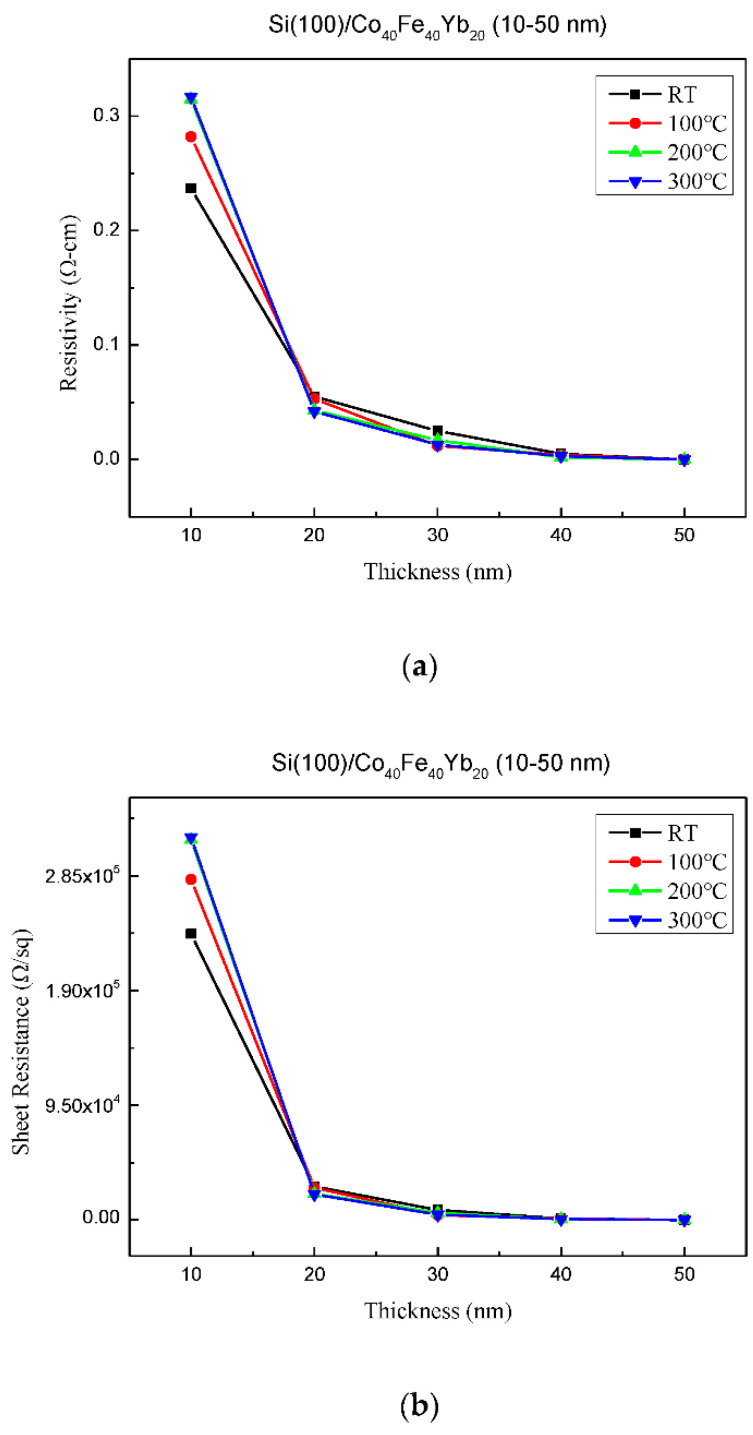

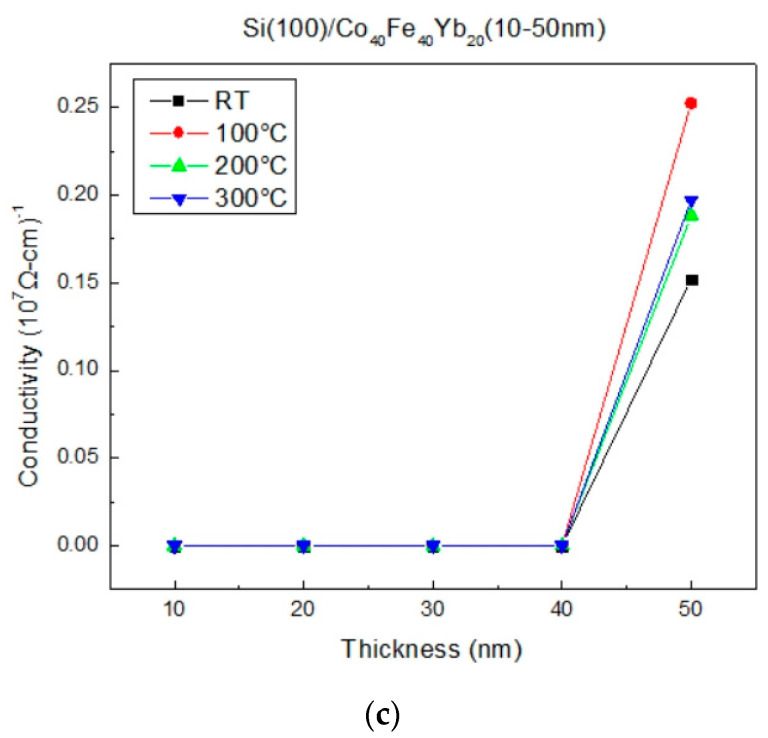
(**a**) Resistivity, (**b**) sheet resistance, and (**c**) conductivity of Co_40_Fe_40_Yb_20_ thin films.

The resistivity and sheet resistance of Co_40_Fe_40_Yb_20_ films under all conditions are depicted in Figure 6a,b. The results confirm that resistance decreased significantly when the thickness and higher annealing temperatures increased due to the oxidation effect of thinner thickness. The electrical resistivity of CoFeYb films varies with changes in carrier concentration and charge carrier mean free path. The thickness of the film is comparable to the mean free path of charge carriers when it is sufficiently thin. It is worth noting that charge carrier collisions with the surface account for a sizable proportion of total collisions [27]. When the thickness is equal to the mean free path of the charge carriers, the resistivity is expected to be thickness dependent. It is also speculated that thicker and higher annealing temperatures have less oxidation effect, reducing the resistance of the current in the flow and reducing the resistivity. Figure 6c illustrates the conductivity of Co_40_Fe_40_Yb_20_ films under all circumstances. From the findings, it can be also indicated that higher annealed temperatures have a lesser oxidation effect, which increases conductivity and carrier mobility while decreasing resistance [28].

### 3.4. Hardness Analysis

**Figure 7:**
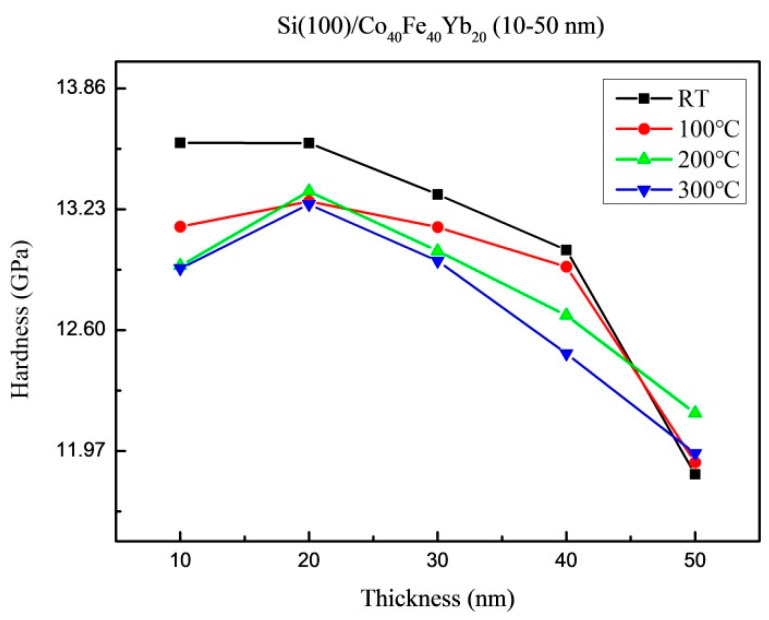
Average hardness of Co_40_Fe_40_Yb_20_ thin films.

The hardness of Co_40_Fe_40_Yb_20_ films under all conditions is shown in Figure 7. The hardness ranged between 11.84 GPa and 13.57 GPa. As-deposited and thinner films have higher hardness than annealed films because more oxidation effect makes dislocations difficult to move and causes strong mechanical characteristics [29]. With thicker thickness, the hardness exhibits a decreasing tendency. Due to its excessive thinness, the thickness is influenced by the substrate, and the stress change it causes is inversely correlated with the film’s thickness. The loading and unloading curves can be used to calculate hardness using the Pharr-Oliver method, which reveals the combined hardness of the silicon substrate and CoFeYb films [30]. As the CoFeYb layer is so thin, it is reasonable to assume that a substrate effect must exist in the nanoindentation measurement [31,32].

### 3.5. Magnetic Analysis

**Figure 8:**
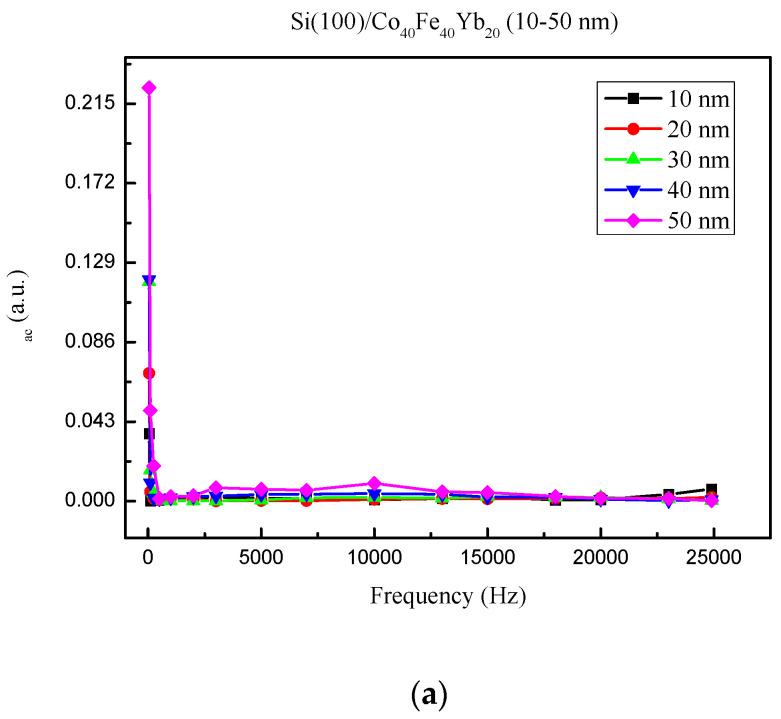

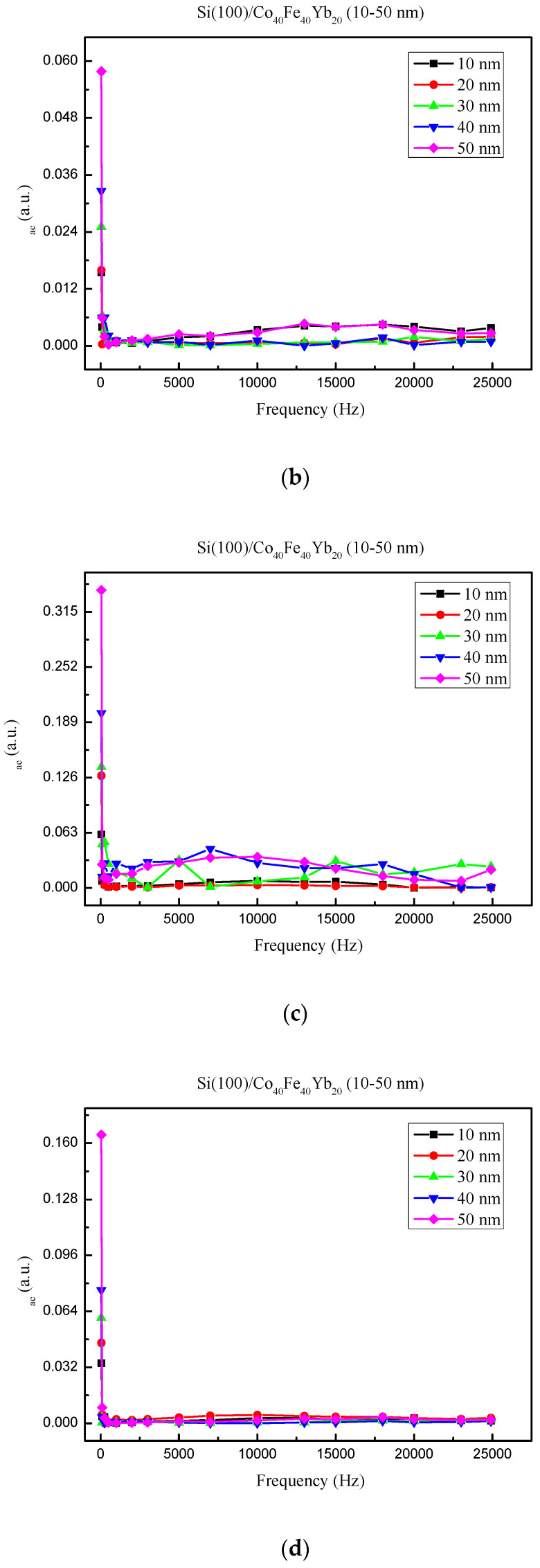
The frequency dependence of alternate-current magnetic susceptibility (χ_ac_) from 50 to 25,000 Hz. (**a**) As-deposited, (**b**) post-annealing at 100 °C, (**c**) post-annealing at 200 °C, and (**d**) post-annealing at 300 °C.

The low-frequency alternative-current magnetic susceptibility (χ_ac_) is displayed in Figure 8a–d under four different conditions. The value of χ_ac_ decreases with frequency in the low-frequency range of 50–25,000 Hz. The outcomes also demonstrate that as film thickness is raised, the associated χ_ac_ value rises. Figure 8 illustrates the sharp frequency reduction in the χ_ac_ values. Furthermore, the as-deposited films revealed a maximum χ_ac_ value of 0.224 at 50 nm. Meanwhile, post-annealing 100 °C films had a maximum χ_ac_ value of 0.058 at 50 nm, while post-annealing 200 °C films had a maximum χ_ac_ value of 0.340 at 50 nm. The maximum χ_ac_ value was 0.165 at 50 nm in the post-annealed 300 °C films.

**Figure 9:**
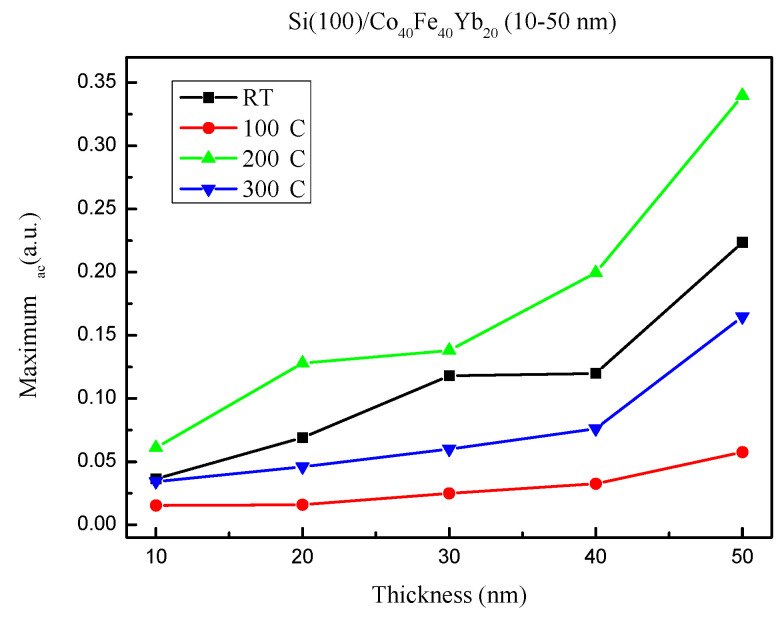
Maximum alternate-current magnetic susceptibility.

Figure 9 depicts the maximum χ_ac_ values for various thicknesses under four conditions. The maximum χ_ac_ value increases as the thickness increases due to the thickness effect [33]. Due to the annealing treatment and less oxidation effect, the highest χ_ac_ value was 0.340 at post-annealing 200 °C with 50 nm. Moreover, the χ_ac_ of as-deposited is larger than 100 °C and 300 °C, owing to magneto-crystalline anisotropy [34,35].

Table 1 displays the maximum χ_ac_ for the optimal resonance frequency (f_res_) under four different conditions. The highest spin sensitivity is present in the maximum χ_ac_ at the ideal resonance frequency [36]. At each thickness, the f_res_ was 50 Hz and calculated to be less than 500 Hz. As a result, it has the potential to be used in low-frequency magnetic applications.

**Figure 10:**
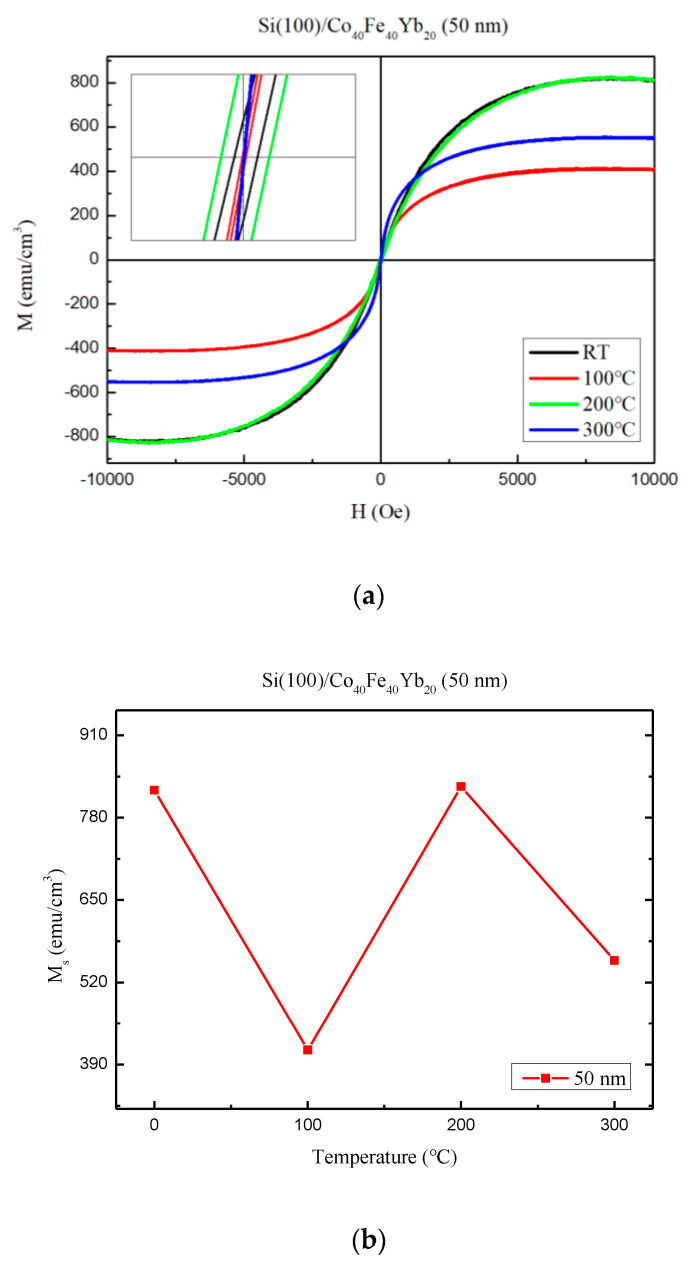
(**a**) In-plane magnetic hysteresis loops of Co_40_Fe_40_Yb_20_ film at 50 nm. (**b**) Saturation magnetization (M_S_) of Co_40_Fe_40_Yb_20_ film at 50 nm.

Because it has the maximum χ_ac_ at 50 nm, it discusses its magnetic characteristics at different annealed temperatures. The magnetic hysteresis loop of the Co_40_Fe_40_Yb_20_ films is shown in Figure 10a for four conditions at 50 nm. For observing the saturated magnetic spin state, an in-plane external magnetic field (H_ext_) of 10 kOe is sufficient. An increased version of the figure shows a low Hc, which in Co_40_Fe_40_Yb_20_ films indicates a soft magnetic characteristic. Figure 10b depicts the Ms of Co_40_Fe_40_Yb_20_ films at 50 nm with various treatments. The maximum value of Ms is found to be at post-annealing 200 °C with 50 nm, which is consistent with χ_ac_. The Ms and χ_ac_ annealed at 300 °C with 50 nm are smaller than 200 °C due to thermal disturbance and more oxidation effect. When the post-annealing is at 100 °C, the Ms significantly decreases in the phenomenon, mainly due to the compensation temperature effect [37].

## 4. Conclusions

In conclusion, the XRD patterns indicate that the Co_40_Fe_40_Yb_20_ films have crystalline CoFe(110), Yb_2_O_3_, and Co_2_O_3_ oxide peaks. Due to insufficient time at low-temperature annealing, the trend of grain size CoFe(110) decreases as the annealing temperature is raised. The contact angles are less than 90°, a characteristic of the hydrophilic film. The best surface energy is 36.45 mJ/mm^2^ at RT with 40 nm. When the thickness is raised, the resistivity and sheet resistance considerably decrease. The maximum hardness is 13.57 GPa at RT with 10 nm. The maximum M_S_ is at post-annealing 200 °C of 50 nm, which is consistent with χ_ac_. The Ms and χ_ac_ annealed at 300 °C with 50 nm are smaller than 200 °C due to thermal disturbance and more oxidation effect. At this temperature, the M_S_ and χ_ac_ values are the highest, which is appropriate for magnetic storage devices. Additionally, this study discovered that the film may be employed in MRAM and recording heads as well as a free layer of the MTJ. The proportion of oxides increased with as-deposited and thinner thicknesses. As a result, the intensity of oxide peaks decreased with increased thickness and annealed temperature, and the weakening oxide peak may reduce interference and enhance electrical and magnetic properties. The ideal condition was found to be 50 nm with annealing at 200 °C because of the high χ_ac_, high Ms, and low resistivity.

## Figures and Tables

**Table 1:** The optimal resonance frequency for various thicknesses of films.

Thickness (nm)	As-Deposited Optimal Resonance Frequency (Hz)	Post-Annealing at 100 °C of Optimal Resonance Frequency (Hz)	Post-Annealing at 200 °C of Optimal Resonance Frequency (Hz)	Post-Annealing at 300 °C of Optimal Resonance Frequency (Hz)
10	50	50	50	50
20	50	50	50	50
30	50	50	50	50
40	50	50	50	50
50	50	50	50	50

## Data Availability

Not applicable.
